# Can we explore AF–pacemakers’ relationship using clinical and echocardiographic parameters in patients with permanent pacemaker? (Echocardiography and subclinical AF in permanent pacemaker)

**DOI:** 10.1007/s10554-022-02719-4

**Published:** 2022-08-26

**Authors:** Ibtesam I. EL-Dosouky, Ahmed Shafie Ammar, Islam A. El Sherbiny, Mohamed M. Mahmoud

**Affiliations:** Cardiology Department, Faculty of Medicine, Zagazig University, Zagazig, 44519 Egypt

**Keywords:** Atrial fibrillation, Pacemaker, Left ventricular stiffness, Diastolic wall strain

## Abstract

Patients on implanted permanent pacemakers frequently develop atrial fibrillation (AF). We aimed to determine the Echocardiographic and clinical parameters predicting AF in patients with a dual-chamber (DDD) pacemaker. This retrospective study included 208 patients with permanent pacemaker, classified according to development of AF during follow up into 2 groups: AF (77, 37%) and non AF (131, 63%), baseline: clinical, ECG(P-wave dispersion) and echo {diastolic wall strain (DWS),left arial volume index (LAVI), left ventricular stiffness index(LVSI)} data were assessed. AF group were older with more P wave dispersion, lesser DWS, greater LVSI& LAVI, LVSI at a cut off > 0.13 and DWS at a cut off < 0.34 were predictors of AF in patients with DDD pacemakers. LVSI and DWS could be used as simple good predictors for AF in patients with DDD pacemakers, for timely initiation of anticoagulants according to CHA_2_DS_2_VASc score to decrease ischemic stroke burden.

## Introduction

Atrial fibrillation (AF) is the commonest sustained cardiac arrhythmia that increases with aging, associated with increased risk of morbidity and mortality. It may be associated with all types of cardiovascular disorders; no single mechanism is concerned with the development of AF. Its pathophysiology may involve a complex interplay of electrophysiologic factors and structural changes. Clinical entities, such as hypertension, heart failure, and coronary disease, through mechanisms such as myocardial stretch, fibrosis, disruption in cell-to-cell coupling, and autonomic dysfunction all may promote development of AF [1]. Up to a third of AF patients are asymptomatic and ischemic stroke is still often the first presentation of AF [2, 3]. This is numerically smaller than patients with clinical AF and the role of oral anticoagulants (OAC) is not yet established [4]. The AF burden and episode duration that merits anticoagulation is also unknown [5].

Patients on implanted permanent pacemakers frequently develop atrial high-rate events (AHREs); defined as events with an atrial frequency of ≥ 180 bpm and a duration of ≥ 5 min (6 min exclude most episodes of over sensing), which is considered as clinically relevant according to current guidelines [6].AHREs are associated with an increased risk of clinical atrial fibrillation (AF) [7], ischemic stroke or systemic embolism [8], and cardiovascular death [9]. AF frequently observed with progressively increased burden over time after implantation [10]. So it is of an interest to explore AF- pacemakers’ relationship [7].

Atrial-based pacing is as close as possible to a physiological pacing method; ventricular pacing is more non-physiological and causes cardiac mechanical dysynchrony. Higher atrial pacing rate by virtue of pacing alone may maintain uniform atrial rate and suppress premature atrial contractions, prevent short-long-short sequences and may prevent bradycardia-induced dispersion of atrial repolarization. Therefore it is conceivable to prevent AF virtually by pacing atria at high rates [11, 12].

### Aim of the study

The present study aimed to determine the echocardiographic and clinical parameters predicting the occurrence of AF in patients with a dual-chamber (DDD) pacemaker, without previously documented AF and to detect the high risk groups vulnerable for AF.

## Methods

This retrospective study (obtained from data base from 2016 to 2021 in the electrophysiology unit) was performed in Cardiology department, Faculty of Medicine, Zagazig University Hospitals, included 208 consecutive patients who had dual-chamber (DDD) pacemaker for sinus node dysfunction (SND) or atrioventricular block (AVB), according to the indication guidelines. Patients were excluded from the study if they had: < 18 years old, no follow-up data/Echocardiographic findings, undergone cardiac surgery, prior clinical atrial tachyarrhythmia (AF or atrial flutter rhythm), significant valvular disease, decompensate renal or liver diseases, uncontrolled thyroid disorders, COPD or pulmonary diseases. the study has been approved by the local ethics committee.

They were classified according to development of AF during follow up into 2 groups:

- Group (A): patients who developed AF episodes on follow up (77 patients; 37%).
- Group (B): patients without AF (131 patients; 63%).

All patients subjected (at base line before pacemaker implantation) to the following:

*Complete history taking*: concerning the basic demographic information including age, gender and risk factors to assess the CHA_2_DS_2_VASc score.

*Physical examination*: Full general and local examination.

## Diagnostic tools

### Laboratory tests

Serum creatinine, creatinine clearance, HbA1C and Hb level.

### Electrocardiography

12-lead electrocardiography (ECG) analysis for; heart rate, PR interval, P wave dispersion (the difference between the longest and the shortest P-wave duration recorded from multiple different-surface ECG leads**, **[13] QRS, QT and corrected QT durations (QTc, using Bazett's method).

### Echocardiography

Transthoracic echocardiography (TTE) for; left atrial volume (LAV) indexed to the BSA for the LAVI, left ventricular ejection fraction (LV EF) and LV mass index (LVMI; using modified Simpson's method) by 2D- echo. The LV diameters were assessed using M-mode in the long axis left parasternal view. Mitral E and A wave velocities and E wave deceleration time were measured using pulsed wave Doppler. Tissue Doppler study for mitral (e') wave velocities (lateral, septal and average) and E/e' ratio was calculated.

LV stroke volume (SV) was measured; (LV end diastolic volume-LV end systolic volume) and indexed to BSA for SV index (SVI).

Diastolic wall strain (DWS) estimated from the following equation: DWS = (PWs-PWd)/PWs; reported as a non-invasive direct measure of the LV compliance [14].

LV stiffness index was measured using the following equation:

$${\text{LV}}\,{\mkern 1mu} {\text{Stiffness}}\,{\text{index}} = {\mkern 1mu} \frac{{{\text{Transmitral}}\,{\mkern 1mu} {\text{E}}:{\mkern 1mu} \,{\text{lateral}}\,{\mkern 1mu} {\text{mitral}}\,{\mkern 1mu}\,{\text{annular}}\,{\mkern 1mu} {\text{e}}^{\prime } }}{{{\text{End}}\,{\mkern 1mu} - {\mkern 1mu} {\text{diastolic}}\,{\mkern 1mu} \,{\text{volume}}}}$$

This parameter provides an estimate of the pressure to volume relationship [15].

## Results

The main demographic data of the study groups are in Table 1, with the mean age of the studied cases 61.67 ± 8.13 years and 53.4% of them were female, 87 with SND and 121 with AVB, AF group was older (p = 0.02), hypertension (HTN) was the most common risk factor found among the studied cases (42.3%) with no significant difference between the studied groups regarding presence of HTN.

**Table 1:** Demographic, clinical, medical and laboratory data of the studied groups

Variable	Total (n = 208)	AF (n = 77)	Non AF (n = 131)	P
Age: (years)	61.67 ± 8.13	63.34 ± 7.91	60.69 ± 8.13	0.02
Serum creatinine (mg/dl)	1.18 ± 0.26	1.21 ± 0.25	1.17 ± 0.27	0.37
Creatinine clearance (ml/min)	67.14 ± 7.84	66.87 ± 7.9	67.29 ± 7.83	0.71
HBA1C (%)	5.99 ± 0.33	6.03 ± 0.32	5.96 ± 0.32	0.18
Hb (g/dl)	12.63 ± 1.11	12.56 ± 1.14	12.67 ± 1.09	0.49
CHA_2_DS_2_-VASc Score:	1.59 ± 1	1.74 ± 0.97	1.5 ± 1.01	0.09

Variable	No	%	No	%	No	%	P
Gender							
Female	111	53.4	42	54.5	69	52.7	0.79
Male	97	46.6	35	45.5	62	47.3	
Risk factors							
CHF	0	0	0	0	0	0	
HTN	88	42.3	34	44.2	54	41.2	0.98
DM	37	17.8	14	18.2	23	17.6	
Vascular insult							
MI	5	2.5	3	3.9	2	1.5	
PVD	8	3.85	2	2.6	6	4.6	
Stroke/TIAs	13	6.3	7	9	6	4.6	
Drugs							
Antiplatelet	63	30.3	23	29.9	40	30.6	0.02
Anticoagulant	8	3.9	5	6.5	3	2.3	
ACEI/ARBS	77	37.1	28	36.4	49	37.5	
BBs	33	15.9	20	26	13	10	
Statins	47	22.6	18	23.4	29	22.2	
Diuretics	21	10.1	10	13	11	8.4	

*SD* Standard deviation: Independent t test *χ*^2^ Chi square test *NS* Non-significant (P > 0.05), *SBP* Systolic Blood pressure, *DPB* Diastolic blood pressure, *HbA1C* glycosylated hemoglobin, *CHF* congestive heart failure, *HTN* Hypertension, *DM* diabetes mellitus, *MI* myocardial infarction, *PVD* peripheral vascular disease, *TIAs* transient ischemic attacks, *BB* beta blockers, *ACEI* angiotensine converting enzyme inhibitor, *ARBS* angiotensin receptor blockers

Anticoagulants, BBs and diuretics were more used in AF group, the mean CHA_2_DS_2_VASc score among the studied cases was 1.59, AF group had higher score but not reach statistical significance (p = 0.09).

P wave dispersion was higher in AF group compared to non AF (45.51 ± 3.33 vs. 20.91 ± 5.77 ms, respectively, p < 0.001), Table 2.

**Table 2:** ECG and Echo results among the studied groups

Variable	AF (n = 77)	Non AF (n = 131)	P
Rate: (beat/min)	64.61 ± 4.04	65.38 ± 3.9	0.18
PR interval: (msec.)	143.91 ± 36.86	145.65 ± 38.77	0.52
P wave dispersion (msec.)	45.51 ± 3.33	20.91 ± 5.77	< 0.001
QRS: (msec.)	150.91 ± 17.33	148.7 ± 18.58	0.40
QTc: (msec.)	469.35 ± 34.58	465.04 ± 36.82	0.41
LA VI (ml/m^2^)	40.65 ± 11.24	28.34 ± 6.07	< 0.001
EDD (mm):	42.96 ± 5.45	43.5 ± 5.89	0.52
ESD (mm):	22.96 ± 3.3	22.59 ± 3.06	0.43
PWs (mm):	12 ± 1.7	13.6 ± 1.2	< 0.001
PWd (mm):	8.7 ± 1.5	8.4 ± 1.2	0.11
DWS:	0.28 ± 0.08	0.38 ± 0.10	< 0.001
EDV (ml):	128.64 ± 5.59	154.81 ± 11.99	< 0.001
ESV (ml):	54.8 ± 3.74	53.52 ± 7.59	0.11
SV I: (ml/m^2^)	43.43 ± 3.88	59.61 ± 9.12	< 0.001
EF: (%)	57.78 ± 3.39	65.56 ± 6.1	< 0.001
E/A ratio	1.13 ± 0.12	1.20 ± 0.16	0.2
E/é	12.19 ± 0.83	6.3 ± 0.98	< 0.001
LV mass index (g/m^2)^	63.87 ± 7.47	64.34 ± 12.08	0.73
LV stiffness (ml^−1^)	0.14 ± 0.01	0.06 ± 0.01	< 0.001

*QTc* corrected QT interval, *msec* millisecond, *DWS* diastolic wall strain assessed by PWs-PWd/PWs, E/é; ratio between early mitral inflow velocity and mitral annular early diastolic velocity(tissue Doppler), *EDD* end diastolic diameter, *ESD* end systolic diameter, *EDV* end diastolic volume, *ESV* end systolic volume, *EF* ejection fraction, *LA* left atrium, *LV* left ventricle, *PWd* Post wall diameter at end-diastole, *PWs* Post wall diameter at end-systole, *SV* stroke volume

AF group compared to non AF group; had a highly significant increase in LAVI (40.65 ± 11.24 vs. 28.34 ± 6.07 ml/m^2^), E/é (12.19 ± 0.83 vs. 6.3 ± 0.98) and LVSI (0.14 ± 0.01vs. 0.06 ± 0.01 ml^−1^), p < 0.001, and a highly significant decrease in posterior wall thickness in systole (PWs); (12 ± 1.7 vs. 13.6 ± 1.2 mm), DWS (0.28 ± 0.08 vs. 0.38 ± 0.10), EDV(128.64 ± 5.59 vs. 154.81 ± 11.99 ml), SVI (43.43 ± 3.88 vs. 59.61 ± 9.12 ml/m^2^) and EF (57.78 ± 3.39 vs. 65.56 ± 6.1%), p < 0.001, Table 2.

LVSI had significant + ve correlation with older age (r = 0.643, p = 0.03), LAVI ( r = 0.928, p < 0.001) and P wave dispersion(r = 0.946, p < 0.001) and had significant − ve correlation with end diastolic volume (EDV), r = − 0.937, p < 0.001), SVI (r = -0.741, p < 0.001), EF (r = − 0.429, p < 0.001) and DWS (r = − 0.92, p < 0.001) among AF cases.

DWS had significant –ve correlation with LAVI (r = − 0.888, p < 0.001), P wave dispersion(r = − 0.94, p < 0.001), and LVSI (r = − 0.600, p < 0.001) and significant + ve correlation with age (r = 0.3, p < 0.001), EDV(r = 0.6, p = 0.001), SVI (r = 0.95, p < 0.001) and EF(r = 0.65, p < 0.001) among AF group.

Univariate analysis indicated that reduced DWS, EDV and SVI, older age, larger LAVI, higher P wave dispersion and increased LVSI were significant variables. On multivariable analysis, only the LVSI (B = − 0.159, HR = 1.5, 95% CI 2.301–0.637, p < 0.001) and DWS (B = 0.165, HR = 0.8, 95% CI 0.323–1.194, p < 0.001) were independent predictors of AF occurrence in patients with dual chamber (DDD) pacemakers, Table 3.

**Table 3:** Multivariate regression analysis for independent predictors of AF, after the implantation of dual-chamber (DDD) pacemaker

Variable	Univariate		Multivariate				
	t	P value	ß	t	HR	95% CI	P value
Age (years)	2.29	**0.02**	− 0.128	− 2.809	0.1	0.013–0.002	0.7
P–wave dispersion (msec)	39.1	** < 0.001**	− 0.245	− 7.664	0.1	0.012–0.007	0.08
CHA_2_DS_2_-VASc Score:	1.66	0.09	0.030	0.652	0.1	0.029–0.058	0.515
EDD (mm)	0.65	0.52	0.034	2.237	0.1	0.020–0.045	0.3
EDV (ml)	− 21.34	< 0.001	− 0.059	− 0.659	0.1	0.007–0.004	0.5
SVI (ml/m^2^)	− 17.72	< 0.001	0.456	9.600	0.1	0.009–0.014	0.1
LAVI (ml/m^2^)	10.26	< 0.001	− 0.173	− 3.286	0.1	0.013–0.003	0.04
LV stiffness (ml^−1^):	50.97	< 0.001	− 0.159	− 3.482	1.5	2.301–0.637	< 0.001*
DWS	7.86	< 0.001	0.165	3.436	0.8	0.323–1.194	< 0.001*
Serum creatinine (mg/dl)	0.9	0.37	− 0.004	− 0.092	0.2	0.153–0.140	0.927
HbA_1_C (%)	1.35	0.18	0.00	− 0.009	0.2	0.124–0.123	0.993
CRP (mg/L)	0.013	1	0.025	0.607	0.1	0.015–0.028	0.544

*t* Independent t test, *HR* hazard ratio, *CI* Confidence interval

**Fig. 1:**
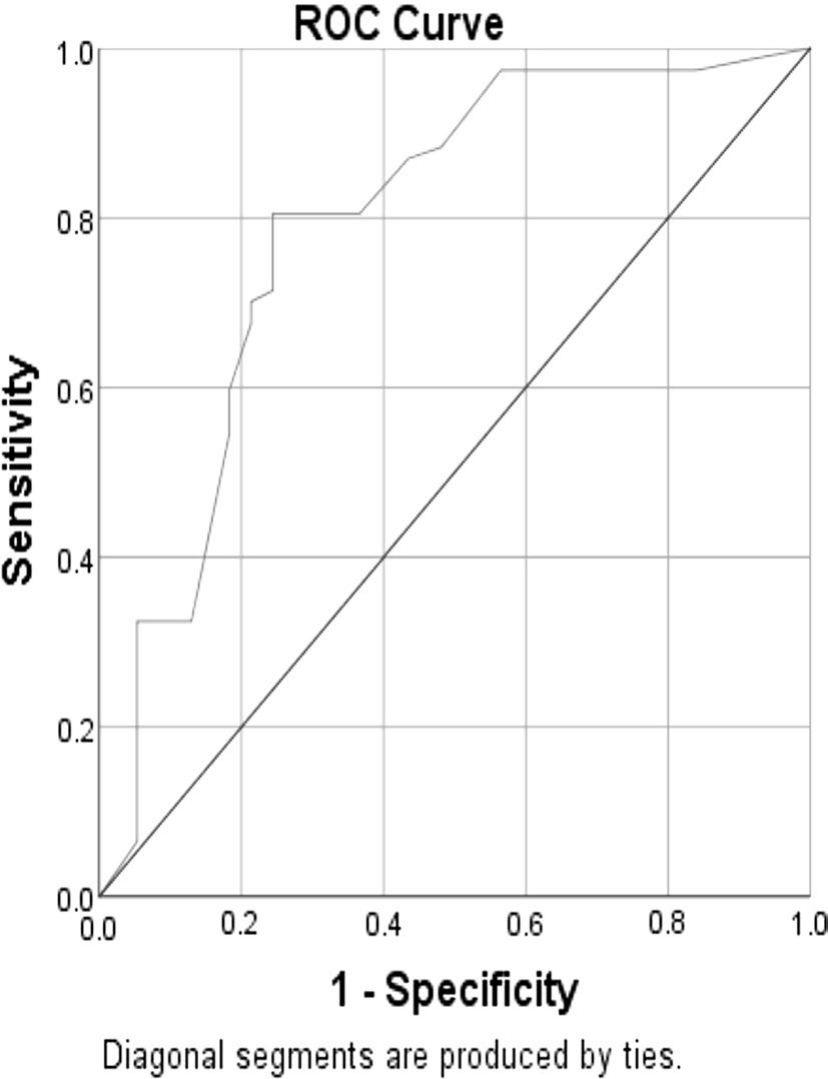
Roc curve of validity of DWS in prediction of AF among the studied cases

**Fig. 2:**
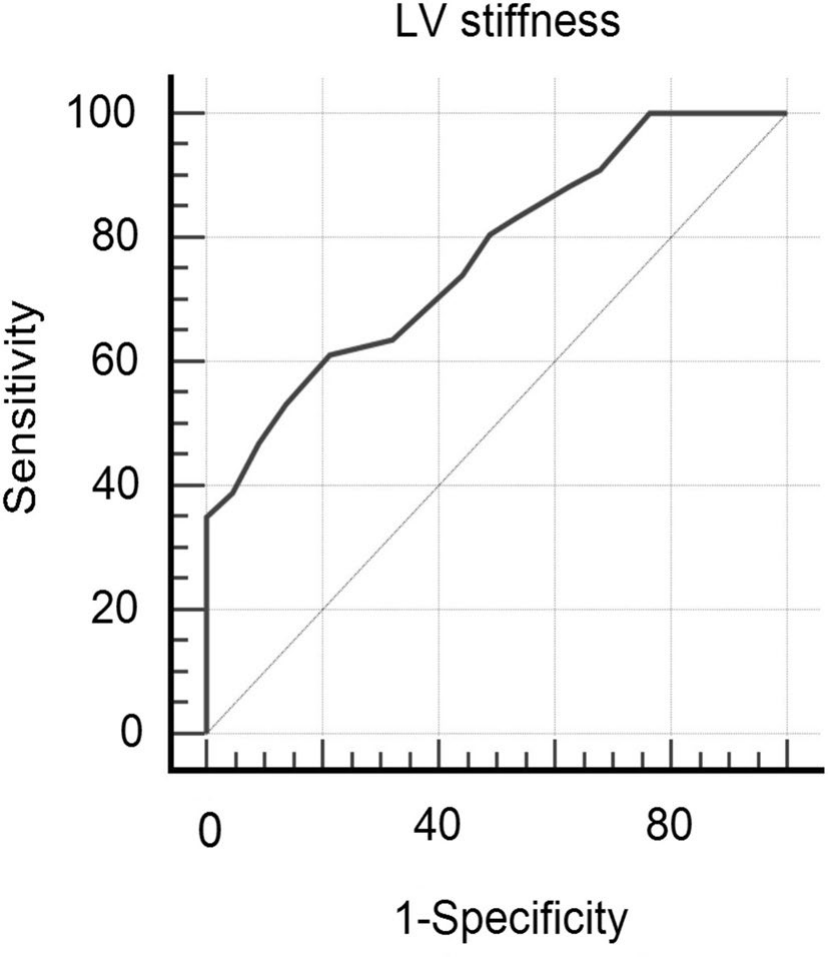
Roc curve of validity of LVSI in prediction of AF among the studied cases

ROC analysis showed that: DWS at a cut off value < 0.34 had a sensitivity of 80.5%, a specificity of 75.6%, NPV of 86.8%, PPV of 66% and an accuracy of 77.4% (AU = 0.79, p < 0.001) in prediction of AF among the studied cases, Fig. 1, and LV stiffness at a cut off value > 0.13 had a sensitivity of 61.1%, a specificity of 78.6%,NPV of 77.4%, PPV of 62.7% and an accuracy of 69.95% (AU = 0.78, p < 0.001) in prediction of AF among the studied cases, Fig. 2.

## Discussion

In an aging patient population, it is frequently necessary to implant a permanent pacemaker. Current dual-chamber permanent pacemakers (PPMs) that incorporate atrial leads are able to detect and store occurrence of arrhythmia as AF [16], most of these episodes were asymptomatic(subclinical episodes), which results in a significant underestimation of the incidence of AF [17] with increased risk of strokes [18].

The link between echocardiographic parameters, clinical data of patients on permanent pacemakers and occurrence of AF need more extensive studies.

LV diastolic wall strain (DWS) can identify a subgroup of subtle LV diastolic dysfunction, as low DWS indicates increased LV stiffness [19]. LV stiffness index is a new echocardiographic index that was found to be accurate measure of LV stiffness [15]. LV stiffness increases in early stages of LV diastolic dysfunction even before abnormal LV relaxation becomes apparent, these in turn augment the LV filling pressure at rest or with exercise, raising the LA wall stress, with atrial structural and electrical remodeling that promotes AF. [20].

In this study we found an increase in the LV stiffness assessed by lower DWS and higher LV stiffness index in AF group, in agreement with many studies [14, 19–22]; the DWS at a cut off value < 0.34 had a sensitivity of 80.5%, a specificity of 75.6%, NPV of 86.8%, PPV of 66% and an accuracy of 77.4% (AU = 0.79, p < 0.001) and the LV stiffness index at a cut off value > 0.13 had a sensitivity of 61.1%, a specificity of 78.6%, NPV of 77.4%, PPV of 62.7% and an accuracy of 69.95% (AUC = 0.78, p < 0.001) in prediction of AF among the studied cases, with significant + ve correlation of the LV stiffness index and other parameters of LV diastolic dysfunction as older age, LAVI and more P wave dispersion, all these parameters were more prominent in AF group, in agreement with Antoni et al. [23], Dilaveris and Gialafos [24] and Lopes RD, et al. [25], while the DWs had significant-ve correlation with LAVI, P wave dispersion and DWS.

We found that EDV and SVI had –ve correlation with LV stiffness index and + ve correlation with DWS, in agreement with Ngiam et al. [26] who stated that the higher the LV stiffness, the lower EDV and SVI. On multivariable analysis we found that AF was only associated with reduced DWS and increased LV stiffness index, which are contributing factors in subtle diastolic dysfunction, patients with reduced DWS (< 0.33) and high LV stiffness index (> 0.13 ml^−1^) had a higher risk to develop AF.

Rovaris et al. [27] indicated that the incidence of AF increased with increasing CHA_2_DS_2_-VASc score, in agreement with these findings, we found that patients in AF group had higher CHA_2_DS_2_-VASc score but not reached significance.

Other parameters of LV diastolic dysfunction as E/é ratio was higher in the AF group in our study in agreement with H. Kishima et al. [21].

Asymptomatic AF delays clinical diagnosis, which can result in ischemic stroke or other embolic complications, so early detection of asymptomatic AF and timely initiation of anticoagulants according to the CHA_2_DS_2_-VASc score are essential for management of this group of patients.

## Conclusion

High LV stiffness index at a cut off value > 0.13 and Low DWS at a cut off value < 0.34 could be used as simple good predictors for development of AF in patients with dual chamber (DDD) pacemaker implantations, for timely initiation of anticoagulant therapy according to CHA_2_DS_2_-VASc score, for patients who develop asymptomatic AF to prevent thromboembolic events and decrease ischemic stroke burden.

### Limitations of the study

This is a retrospective study only from the same geographic area and from a single medical center (Zagazig University Hospitals) with a small number of patient subgroups, cases of cardiac resynchronization therapy (CRT) or ICD implantation were excluded and, thus, the AF detection rate may be underestimated, with lake of information about the incidence of stroke, systemic embolism, heart failure, acute myocardial infarction, and other cardiovascular and cerebrovascular events within the period of pacemaker implantation, in many of our cases the echo report/measurements obtained from the data base.

### Recommendations

LV stiffness index and DWS are simple useful predictors of AF in patients with DDD pacemaker. Our findings may provide additional diagnostic information for the other diagnostic parameters. In our opinions patients with pacemakers detected AHREs, low DWS, high LV stiffness index and high CHA_2_DS_2_-VASc score need oral anticoagulation to prevent thromboembolic events and decrease burden of ischemic stroke as the incidence of subclinical AF is high in this patient population. On follow up of pacemaker, once AHRE occurs, 24-h Holter should be carried out for early AF detection and treatment. Time lapse between pacemaker implantation and development of AF, body mass index and presence of coronary artery disease or if any of AV block events that may be driven by underlying CAD/ACS can improve the results and so the novel markers such as LV/LA strain.

## Data Availability

The data that support the findings of this study are available from the corresponding author upon reasonable request.
